# The predictive value of serum neurofilament light chain levels beyond MRI measures for clinical and radiological disease progression in the earliest stages of multiple sclerosis

**DOI:** 10.1007/s00415-026-14100-9

**Published:** 2026-09-09

**Authors:** Rozemarijn M. Mattiesing, Giordano Gentile, Marco Battaglini, Nicola De Stefano, Iman Brouwer, Eva M. M. Strijbis, Bernard M. J. Uitdehaag, Frederik Barkhof, Hugo Vrenken, Menno M. Schoonheim

**Affiliations:** 1 MS Center Amsterdam, Radiology and Nuclear Medicine, Vrije Universiteit Amsterdam, Amsterdam Neuroscience, Amsterdam UMC, PO Box 7057, 1007 MB Amsterdam, The Netherlands; 2 Department of Medicine, Surgery and Neuroscience, University of Siena, Siena, Italy; 3 Siena Imaging SRL, Siena, Italy; 4 MS Center Amsterdam, Neurology, Vrije Universiteit Amsterdam, Amsterdam Neuroscience, Amsterdam UMC, Amsterdam, The Netherlands; 5 Queen Square Institute of Neurology and Centre for Medical Image Computing, University College London, London, UK; 6 MS Center Amsterdam, Anatomy and Neurosciences, Vrije Universiteit Amsterdam, Amsterdam Neuroscience, Amsterdam UMC, Amsterdam, The Netherlands

**Keywords:** Multiple sclerosis, Neurofilament, Atrophy, Lesions, Progression

## Abstract

**Objective:**

This study investigated the predictive value of neurofilament light chain (NfL) levels beyond MRI measures of disease activity in multiple sclerosis (MS).

**Methods:**

This study is a retrospective post hoc analysis of the early interferon treatment group of the combined 5-year REFLEX/REFLEXION studies in clinically isolated syndrome. Baseline z-scored NfL (zNfL) and changes in zNfL during the first two years were related to brain atrophy, lesion activity, and clinical progression.

**Results:**

This study included 262 patients (61.83% female, mean age 31.68 ± 8.43, median Expanded Disability Status Scale 1.5). Mean zNfL levels were highest at baseline and higher in patients with gadolinium-enhancing lesions or high T2 lesion counts (*p* < 0.001). Baseline zNfL correlated with higher volumes of T2 lesions at baseline and new/enlarging/shrinking/disappearing lesions in years 1 and 2 (*r*_s_ = 0.20–0.68, *p* = 0.002– <0.001), faster subsequent central atrophy (*r* = 0.16, *p* = 0.035), and an increased risk of and faster conversion to clinically definite MS (OR = 1.18, *p* = 0.035; HR = 1.16, *p *= 0.021). Baseline zNfL was no longer predictive when including baseline MRI variables and demographics. Longitudinal zNfL changes correlated especially with shrinking lesions during year 1 (*r*_s_ = − 0.42, *p* < 0.001) and new/enlarging lesions during year 2 (*r*_s_ = 0.26 and 0.23, *p* < 0.001). Subsequent global and central atrophy (*r* = −0.18, *p* = 0.025; *r* = 0.20, *p* = 0.015) were only related to zNfL changes during year 2, even after including baseline MRI variables and demographics.

**Discussion:**

Cross-sectional zNfL was related to active disease and predictive of disease progression, but not after taking MRI measures into account. The longitudinal zNfL changes were related to lesion activity and subsequent atrophy, even after including MRI measures.

**Supplementary Information:**

The online version contains supplementary material available at https://doi.org/10.1007/s00415-026-14100-9.

## Introduction

Neurofilament light chain (NfL) is a neuron-specific cytoskeletal protein that is released upon neuronal injury and can be measured in the CSF and blood. In multiple sclerosis (MS), an immune-mediated and degenerative disorder of the CNS, NfL levels have been mostly considered to reflect acute neuro(axo)nal damage [1]. NfL levels have been shown to relate to disease activity in MS, i.e., T2 lesions, gadolinium-enhancing lesions and relapses, but also to neurodegenerative aspects such as brain and spinal cord volume loss [2, 3]. Serum NfL (sNfL) measurements have been validated with high comparability to CSF [4] and sNfL holds the promise for allowing frequent monitoring in clinical practice, with its low cost and low patient burden [1]. Therefore, sNfL is considered as a potential prognostic biomarker that could also aid in treatment monitoring, clinical decision-making, and stratifying for risk of clinical progression early in the disease course.

This potential is important since predicting disease progression remains difficult, especially early in the course of MS, hampering appropriate treatment optimization [5]. Previous work has shown that clinical and MRI measures can predict disease progression [6], to which sNfL might add predictive value [2, 3]. In addition, it remains unclear whether cross-sectional and longitudinal sNfL levels provide the same or additional information as MRI measures for long-term prediction of disease progression in early MS.

Particularly the predictive value for disease progression of fluctuations in sNfL levels has not been investigated extensively. Previous studies in both early and later MS have shown that longitudinal increases in sNfL were related to disability progression [7–9] and predicted transition to secondary progressive MS [10] and impending relapses [11]. However, others found no such relations in progressive MS [12]. Results for the relationship between longitudinal changes in sNfL and atrophy were inconsistent [7, 13, 14]. As such, most of the literature on the predictive value of sNfL changes over time remains inconsistent and critically understudied, especially in early MS. In the current study, we therefore investigated the relationships of cross-sectional sNfL levels and their change over time with lesion changes and atrophy rates, as well as their added predictive value for radiological and clinical disease progression in patients with clinically isolated syndrome (CIS) at baseline in the REFLEX/REFLEXION studies, in subjects all receiving early treatment with subcutaneous interferon beta-1a. We specifically investigated the period after treatment initiation in active disease and a period where treatment has been stabilized.

## Methods

### Dataset

The 2-year double-blind multicenter clinical trial REFLEX (NCT00404352/EudraCT 2006–002982-38) and its preplanned 3-year extension called REFLEXION (NCT00813709/EudraCT 2008–004954-34; REbif FLEXible dosing in early MS/extensION) assessed the (long-term) impact of subcutaneous interferon beta-1a in clinically isolated syndrome (CIS). The REFLEX/REFLEXION studies were conducted from November 16, 2006, to August 30, 2013 and the procedures and the design of the study have been described in detail previously [15, 16]. As described, patients with CIS within 60 days before study entry at high risk of converting to MS were included and either randomized to one of two early treatment arms, where treatment with sc IFN β−1a 44 µg was initiated once a week or three times a week, or to the delayed treatment arm in which, during the first 2 years (i.e., the REFLEX phase), patients did not receive treatment (placebo group) but at the start of REFLEXION received sc IFN β−1a 44 µg three times a week. If patients converted to clinically definite MS (CDMS), they received open-label treatment with sc IFN β−1a 44 µg three times a week. For the current study, the data of 262 patients [17] with CIS at baseline that received treatment once a week or three times a week at the beginning of the study were used, and together were considered as the early treatment group. The early treatment group was selected for the current study because participants consistently started treatment once or three times a week since the beginning of the REFLEX study. This retrospective post hoc analysis is covered by the ethical approvals of the REFLEX/REFLEXION studies, which were approved by the relevant institutional review board or independent ethics committee of each participating center (see Ethical Approval section for more details).

### Measurements

#### MRI data

Multicenter MRI scans were acquired yearly during a follow-up period of 5 years and consisted of 1 × 1 × 3 mm^3^ 2D T1-weighted pre- and post-gadolinium and 2D dual-echo proton-density-/T2-weighted images. Baseline MRI measurements included: normalized brain volume (NBV, in L, from SIENAX) [18], T2 hyperintense lesion count and volume (T2LV, in mL), and T1 gadolinium-enhancing lesion volume (T1GdLV, in mL). The participants were categorized based on showing at least 9 T2 lesions (high lesion load) at baseline or not; and having at least 1 gadolinium-enhancing lesion (acute inflammation) or not at baseline [19]. The Image Analysis Center of Amsterdam, then at VU University Medical Center, provided the manual lesion outlines.

#### Clinical data

Expanded Disability Status Scale (EDSS) and CDMS assessments were conducted every 3 months for REFLEX and every 6 months for the REFLEXION study.

#### Neurofilament light chain

Serum NfL (sNfL) concentrations were measured from blood samples collected during the REFLEX study. As previously described [20], the baseline, month 6, month 12, and month 24 samples were analyzed using a sensitive single molecule array assay (as described previously [4]) and were run on a Simoa HD-1 instrument (Quanterix, Billerica, MA, USA) using a two-step Assay Neat 2.0 protocol. Age- and body mass index (BMI)-adjusted z-scored sNfL (zNfL) levels were then calculated by bridging the values from the Basel internal ELISA method to Quanterix (based on paired samples) and using a large (Simoa/Quanterix-based) healthy control reference database [21]. zNfL change during year 1 and year 2 of the study was calculated by subtracting the first visit’s value from the second visit’s value.

#### Lesion volume changes

While lesion volume increases reflect inflammatory activity, the meaning of shrinking lesions remains unclear. This possibly reflects a process of repair but also resolving edema, especially during the initiation of treatment as previously explored [22]. As such, lesion volume changes during year 1 and year 2 were quantified and categorized into four categories using our semiautomated method [23] which was applied in our previous work on the same dataset [17, 22, 24]. The four categories were: new, enlarging (i.e., positive lesion activity), disappearing, and shrinking (i.e., negative lesion activity). The volumes of changing voxels were quantified in these four categories as well. Resolving edema for lesions during year 1 was defined as the section of a lesion showing contrast enhancement at baseline within a lesion shrinking in that year. This was quantified in a T2 halfway space between baseline and month 12 using procedures analogous to FSL-SIENA [18, 23]. Transformations were concatenated to prevent extra interpolation steps. Subsequently, the volume (in mL) of the enhancing section of shrinking lesions was quantified by taking the intersection of the gadolinium-enhancing lesion masks (in the year 1 halfway space) and the masks of the lesions that were classified as shrinking during year 1.

#### Atrophy

Global and central atrophy measures during year 1 and year 2 were calculated using FSL-SIENA [18] and FSL-VIENA [25] as percentage brain volume change (PBVC) and percentage ventricular volume change (PVVC), respectively. These measurements were performed after lesion filling as part of our previous work [17, 24]. A more negative value for PBVC and more positive value for PVVC are indicative of faster atrophy, respectively. Subjects were classified as showing pseudoatrophy during year 1 of the study (yes/no) based on a cutoff. The cutoff was defined using a PBVC during year 1 of <−0.5% (based on previous findings) [26] and the presence of shrinking lesion activity (higher than the median change of 0.27 mL in subjects with PBVC < −0.5%).

### Outcome measures

As the clinical impact of radiological disease progression is expected to manifest later, this study aims to predict both clinical and radiological progression, using sNfL as a predictor. Subsequently, it is investigated whether sNfL has additional predictive value beyond clinical and MRI markers. For this reason the next section on outcome measures is divided into two sections.

#### Clinical disease progression

##### (Time to) CDMS

In REFLEX/REFLEXION [15, 16] conversion to CDMS during the 5-year period was defined as a relapse or sustained progression in EDSS score of ≥1.5 points compared with baseline and confirmed at the next visit. Time to CDMS conversion was also recorded.

##### EDSS-based confirmed disability progression

Confirmed disability progression (CDP) during the 5-year period was defined as an increase of 1.5, 1, or 0.5 points on the EDSS for a baseline EDSS of 0, 1–5, or ≥5.5, respectively, at any time compared with baseline and confirmed at the next visit.

#### Radiological disease progression

##### Atrophy during the last three years of the study

As a measure of radiological disease progression, global atrophy (PBVC_m24-m60_) and central atrophy (PVVC_m24-m60_) during the last three years of the study (the REFLEXION period) were also calculated based on the yearly PBVC and PVVC measurements, respectively, as part of our previous study [22] and used in the current analyses.

### Statistical analysis

We investigated the relationship between zNfL and MRI volumetrics to explore the predictive value of zNfL values, how zNfL evolves over time, and how zNfL relates to the different MRI and disease progression measures. An alpha level of 0.05 was used as the cutoff for significance and statistical analyses were performed with IBM SPSS-28.

Using repeated measures ANOVA, we first assessed the evolution of zNfL over time in the entire patient population. Next, we looked at this evolution in different subgroups, based on having a high lesion load or acute inflammation at baseline. When the assumption of sphericity was violated (as indicated by Mauchly’s test), we applied the Greenhouse–Geisser correction.

Next, we evaluated potential cross-sectional and longitudinal relationships using correlation and regression analyses. After identifying potential relationships, these analyses were repeated when relevant to assess whether they would survive confounder correction.

#### Baseline zNfL correlations and regression models

At baseline, we investigated how zNfL was related to T2LV (at baseline and categories of lesion volume change during year 1 and year 2) and resolving edema during year 1 in the subgroup where this was present using Spearman (r_s_) correlations. We also investigated how baseline zNfL was related to brain volume (baseline NBV and PBVC/PVVC during year 1 and year 2) using Pearson (r) correlations. An independent *t*-test was performed to investigate the difference in baseline zNfL between subjects with and without pseudoatrophy during year 1. Welch’s *t*-test was applied when the assumption of equal variances was violated (as indicated by Levene’s test).

The relationship between baseline zNfL and clinical disease progression was investigated by performing logistic (CDMS and EDSS-based CDP) and Cox regressions (time to CDMS). We also investigated the relationship between baseline zNfL and radiological disease progression (PBVC_m24-m60_ and PVVC_m24-m60_) using Pearson correlations. These analyses were also repeated while correcting for potential baseline MRI and demographic confounders, i.e., age, sex, baseline EDSS score (when applicable), baseline NBV, baseline T2LV, and baseline T1GdLV.

Analyses were repeated at year 1, i.e., after everyone has received one year of treatment, to investigate the predictive value of zNfL during a stable treatment period after the initial acute phase.

#### Longitudinal zNfL correlations and regression models

For the following analyses, if relationships between variables were significant, we also corrected for baseline zNfL by using partial Spearman/Pearson correlations and regressions to investigate if the effect remained.

The relationship between concurrent changes in zNfL and categories of lesion volume change during year 1 and year 2, and the volume of resolving edema during year 1 in the subgroup where this was present was determined by calculating Spearman correlations. We also investigated how concurrent changes in zNfL were related to atrophy during year 1 and year 2 by using Pearson correlations.

The relationship between longitudinal changes in zNfL during year 1 and year 2 and clinical disease progression was investigated by performing logistic (CDMS and EDSS-based CDP) and Cox (time to CDMS) regressions. We also investigated the relationship between longitudinal changes in zNfL and radiological disease progression by using Pearson correlations. These analyses were also repeated while correcting for the potential baseline MRI and demographic confounders.

### Data availability

Any requests for data by qualified scientific and medical researchers for legitimate research purposes will be subject to Merck’s (CrossRef Funder ID: https://doi.org/10.13039/100009945) Data Sharing Policy. All requests should be submitted in writing to Merck’s data sharing portal (https://www.merckgroup.com/en/research/our-approach-to-research-and-development/healthcare/clinical-trials/commitment-responsible-data-sharing.html). When Merck has a co-research, co-development, or co-marketing or co-promotion agreement, or when the product has been out-licensed, the responsibility for disclosure might be dependent on the agreement between parties. Under these circumstances, Merck will endeavor to gain agreement to share data in response to requests.

## Results

In the early treatment group consisting of 262 subjects, 105 (40.08%) converted to CDMS and 48 (18.32%) showed EDSS-based CDP during the study period. Baseline descriptives are provided in Table 1. A more detailed description of the cohort is provided in our previous study [22].

**Table 1:** Descriptives of early treatment group

Variables	Baseline
Age (y), mean (SD)	31.68 (8.43)
Sex (female), *N* (%)	162 (61.83%)
EDSS score, median (min, max)	1.5 (0.0, 4.0)
sNfL (pg/mL), median (min, max)	26.62 (1.33, 437.58)/M12: 14.30 (3.20, 283.58)
zNfL, mean (SD)	1.68 (1.70)/M12: 0.29 (1.52)
≥9 T2 lesions	199 (75.95)
At least 1 T1Gd+ lesion	109 (41.60%)
*T1GdLV (mL), median (min, max)*	
Total group Patients with at least 1 T1Gd+ lesion	0.00 (0.00, 2.95) 0.12 (0.01, 2.95)
T2LV (mL), median (min, max)	2.14 (0.02, 25.32)
NBV (L), mean (SD)	1.53 (0.07)

Medians were used for variables that were not normally distributed

*EDSS* Expanded Disability Status Scale, *M12* month 12 visit, *NBV* normalized brain volume, *pg/mL* picograms per milliliter, *SD* standard deviation, *sNfL* serum neurofilament light chain, *T1Gd+* gadolinium-enhancing lesion, *T1GdLV* T1 gadolinium-enhancing lesion volume, *T2LV* T2 hyperintense lesion volume, *y* year, *zNfL* z-scored serum neurofilament light chain

### zNfL levels over time

**Fig. 1:**
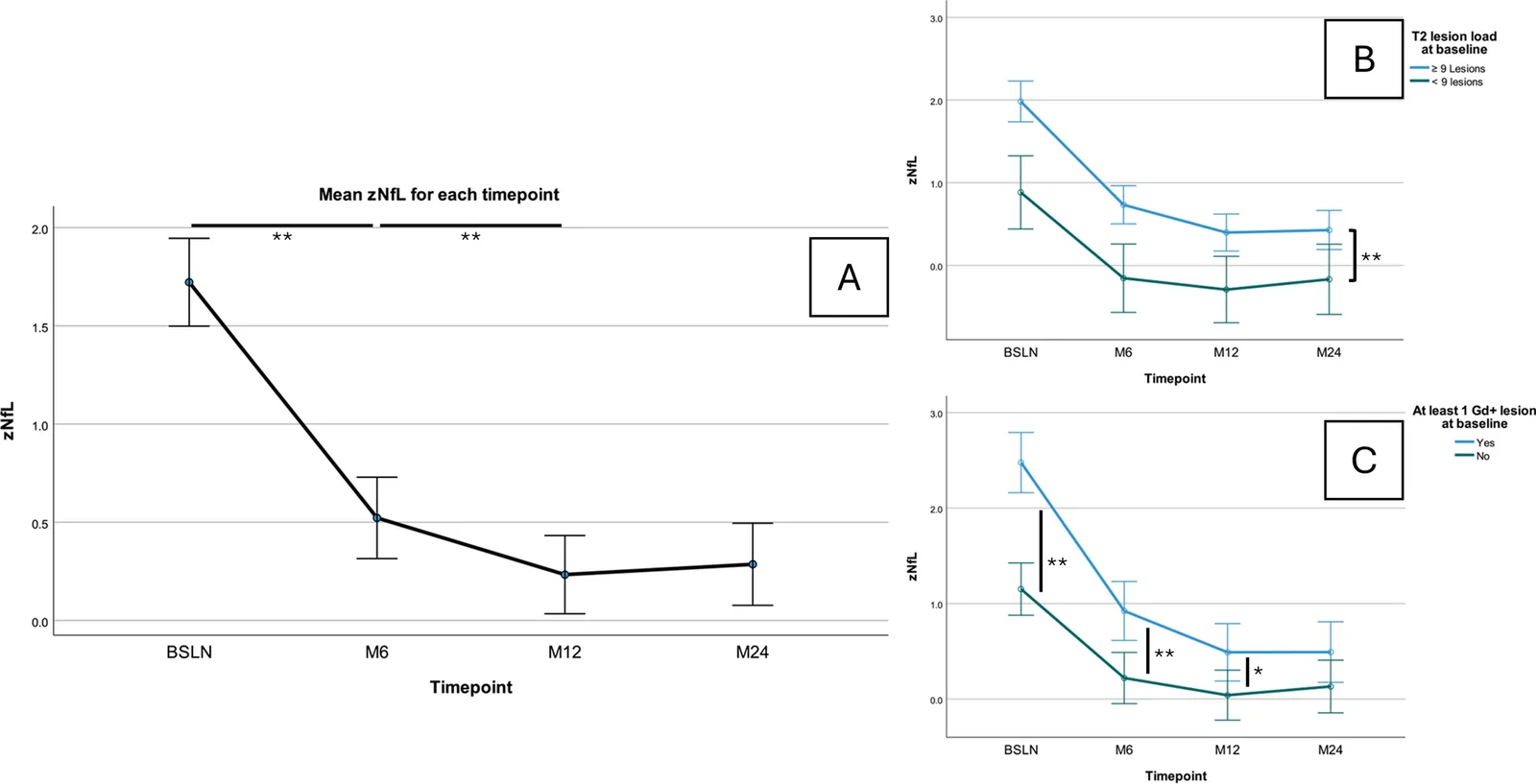
Evolution of z-scored serum neurofilament light chain levels over time. Mean z-scored serum neurofilament light chain (*zNfL*) levels (± 95% confidence interval) over time (**A**) and in different groups (**B** and **C**). **B** subjects with less than or at least 9 lesions at baseline. **C** subjects with and without at least 1 gadolinium-enhancing (Gd +) lesion at baseline. *BSLN* baseline, *M* month; *p < 0.05; **p < 0.001

A repeated measures ANOVA indicated a significant change of zNfL over time (*F*(2.45, 542.84) = 98.73, *p* < 0.001, *η*^2^*p *= 0.31). post hoc pairwise comparisons showed that there was a significant difference between baseline and month 6, and between month 6 and month 12 (both *p* < 0.001). As shown in Fig. 1, Panel A, zNfL levels dropped during the first year of treatment and then remained stable during the second year of the study, which was confirmed by the non-significant post hoc pairwise comparison between month 12 and month 24 (*p* = 0.513).

### zNfL levels over time in different subgroups

Repeated measures ANOVAs indicated that overall, zNfL levels were higher over time in subjects with a high lesion load (greater than or equal to 9 T2 lesions at baseline) compared to subjects with a lower lesion load (*F*(1, 221) = 17.02, *p* < 0.001, *η*^2^*p* = 0.07), and in subjects with at least 1 gadolinium-enhancing lesion at baseline compared to those without (*F*(1, 221) = 17.32, *p* < 0.001, *η*^2^*p* = 0.07). There was a significant interaction between timepoint and the presence of acute inflammation at baseline: *F*(2.52, 555.82) = 9.77, *p* < 0.001, *η*^2^*p* = 0.04). Post hoc pairwise comparisons showed that patients with at least 1 gadolinium-enhancing lesion at baseline had higher zNfL than patients without baseline enhancing lesions; this was observed at baseline and month 6 (both *p* < 0.001), and at month 12 (*p* = 0.027), with a trend for month 24 (*p* = 0.093), which indicates a steeper decline over time for subjects with acute inflammation at baseline. This is illustrated in Fig. 1, Panels B and C.

### Baseline zNfL correlations and regression models

#### Baseline zNfL and lesions

Higher baseline zNfL was related to higher baseline T2LV (*r*_s_ = 0.48, *p* < 0.001), and this relationship was significant both in subjects with at least 1 gadolinium-enhancing lesion at baseline (*r*_s_ = 0.47, *p* < 0.001) and those without (*r*_s_ = 0.40, *p* < 0.001). As shown in Table 2, higher baseline zNfL was related to both higher positive (new and enlarging) and negative lesion activity (disappearing and shrinking), and especially shrinking lesion activity during year 1. We also calculated the resolving edema by taking the volume of baseline gadolinium-enhancing sections of shrinking lesions during year 1, and focused on the subgroup where this was present (*N* = 70, median (min, max) = 0.14 mL (0.01, 2.51)), and higher baseline zNfL was indeed related to higher resolving edema during year 1 (*r*_s_ = 0.49, *p* < 0.001).

**Table 2:** Relationship between baseline zNfL and lesion volume changes during year 1 and year 2

zNfL baseline	NewLVC-y1	EnlLVC-y1	DisLVC-y1	ShrinkLVC-y1
	*r*_s_ = 0.33 *p* < 0.001	*r*_s_ = 0.39 *p* < 0.001	*r*_s_ = 0.34 *p* < 0.001	*r*_s_ = 0.68 p < 0.001
zNfL baseline	NewLVC-y2	EnlLVC-y2	DisLVC-y2	ShrinkLVC-y2
	*r*_s_ = 0.20 *p* = 0.002	*r*_s_ = 0.29 *p* < 0.001	*r*_s_ = 0.27 *p* < 0.001	*r*_s_ = 0.26 *p* < 0.001

*Dis* disappearing, *Enl* enlarging, *LVC* lesion volume change, *y* year, *zNfL* z-scored serum neurofilament light chain

#### Baseline zNfL and brain volume

Baseline zNfL was not related to NBV at baseline. Higher baseline zNfL was related to faster longitudinal global and central atrophy during year 1 and year 2:

PBVC year 1 (*r* = − 0.17, *p* = 0.010), PBVC year 2 (*r* = − 0.18, *p* = 0.005);

PVVC year 1 (*r* = 0.20, *p* = 0.002), PVVC year 2 (*r* = 0.18, *p* = 0.006).

Out of 250 subjects with available data, 59 (22.52%) exhibited pseudoatrophy during year 1 and showed a significantly higher zNfL level at baseline compared to those without pseudoatrophy (mean = 2.78, SD = 1.11 vs mean = 1.30, SD = 1.68; *t*(147.32) = 7.81, *p* < 0.001, *d* = 0.94).

#### Predictive value of baseline zNfL for disease progression

Next we investigated the relationship between baseline zNfL and clinical progression anywhere during the 5-year period. Higher baseline zNfL was significantly related to a higher risk of conversion to CDMS (odds ratio (OR) = 1.18, 95% CI [1.01, 1.38], *p* = 0.035 and a faster time to CDMS (hazard ratio (HR) = 1.16, 95% CI [1.02, 1.31], *p* = 0.021). Baseline zNfL was not significantly related to EDSS progression.

Higher baseline zNfL was significantly related to faster central atrophy during the last three years of the study (*r* = 0.16, *p* = 0.035). As global atrophy only showed a trend for an association with baseline zNfL (*r* = − 0.15, *p* = 0.050), it was not considered further.

After controlling for baseline MRI confounders and demographics, baseline zNfL was no longer a significant predictor of CDMS status (OR = 1.16, 95% CI [0.97, 1.39], *p* = 0.101), time to CDMS (HR = 1.14, 95% CI [0.99, 1.31], *p* = 0.075), or central atrophy during the last three years of the study (*B* = 0.89, 95% CI [− 0.07, 1.84], *p* = 0.069).

### Relationships during stable treatment

Finally, we investigated whether similar effects would be observed when looking at the period after the initial acute phase when treatment has been stabilized (i.e., at month 12). Cross-sectional results at month 12 were similar to baseline, except that the association of zNfL at month 12 with central atrophy during the last three years of the study was not significant (*r* = 0.15, *p* = 0.061).

### Longitudinal zNfL correlations and regression models

#### Concurrent lesion volume changes

##### Year 1

An increase in zNfL during year 1 was related to less negative lesion activity (i.e., shrinking and disappearing lesions) in the same period (especially shrinking lesion activity; see first row of Table 3), and also to less resolving edema in the subgroup where this was present (*r*_s_ = − 0.34, *p* = 0.005). However, after correcting for baseline zNfL, an increase in zNfL during year 1 was related to higher positive lesion activity (i.e., new and enlarging lesions; see second row of Table 3), and the relationship with resolving edema was non-significant (partial *r*_s_ = − 0.16, *p* = 0.212).

**Table 3:** Relationship between changes in zNfL and lesion volumes during year 1

ΔzNfL_bsln-m12_	NewLVC-y1	EnlLVC-y1	DisLVC-y1	ShrinkLVC-y1
	Ns	Ns	*r*_s_ = − 0.18 *p* = 0.006	*r*_s_ = − 0.42 *p* < 0.001
ΔzNfL_bsln-m12_ corrected for baseline zNfL	Partial *r*_s_ = 0.24 *p* < 0.001	Partial *r*_s_ = 0.28 *p* < 0.001	Ns	Ns

*bsln* baseline, *Dis* disappearing, *Enl* enlarging, *LVC* lesion volume change, *ns* not significant, *m* month, *r*_*s*_ Spearman correlation, *y* year, *zNfL* z-scored serum neurofilament light chain

##### Year 2

As shown in Table 4, an increase in zNfL during year 2 was related to more positive lesion activity. This remained the same when correcting for baseline zNfL.

**Table 4:** Relationship between changes in zNfL and lesion volumes during year 2

ΔzNfL_m12-m24_	NewLVC-y2	EnlLVC-y2	DisLVC-y2	ShrinkLVC-y2
	*r*_s_ = 0.26 *p* < 0.001	*r*_s_ = 0.23 *p* < 0.001	Ns	Ns
ΔzNfL_m12-m24_ corrected for baseline zNfL	Partial *r*_s_ = 0.27 *p* < 0.001	Partial *r*_s_ = 0.26 *p* < 0.001	Ns	Ns

*Dis* disappearing, *Enl* enlarging, *LVC* lesion volume change, *ns* not significant, *m* month, *r*_*s*_ Spearman correlation, *y* year, *zNfL* z-scored serum neurofilament light chain

#### Concurrent brain volume changes

zNfL changes during year 1 and year 2 were not significantly related to atrophy within the same year.

#### Predictive value for disease progression

zNfL changes during year 1 and year 2 were not significantly related to any of the clinical disease progression measures (CDMS status, time to CDMS, and EDSS progression).

**Fig. 2:**
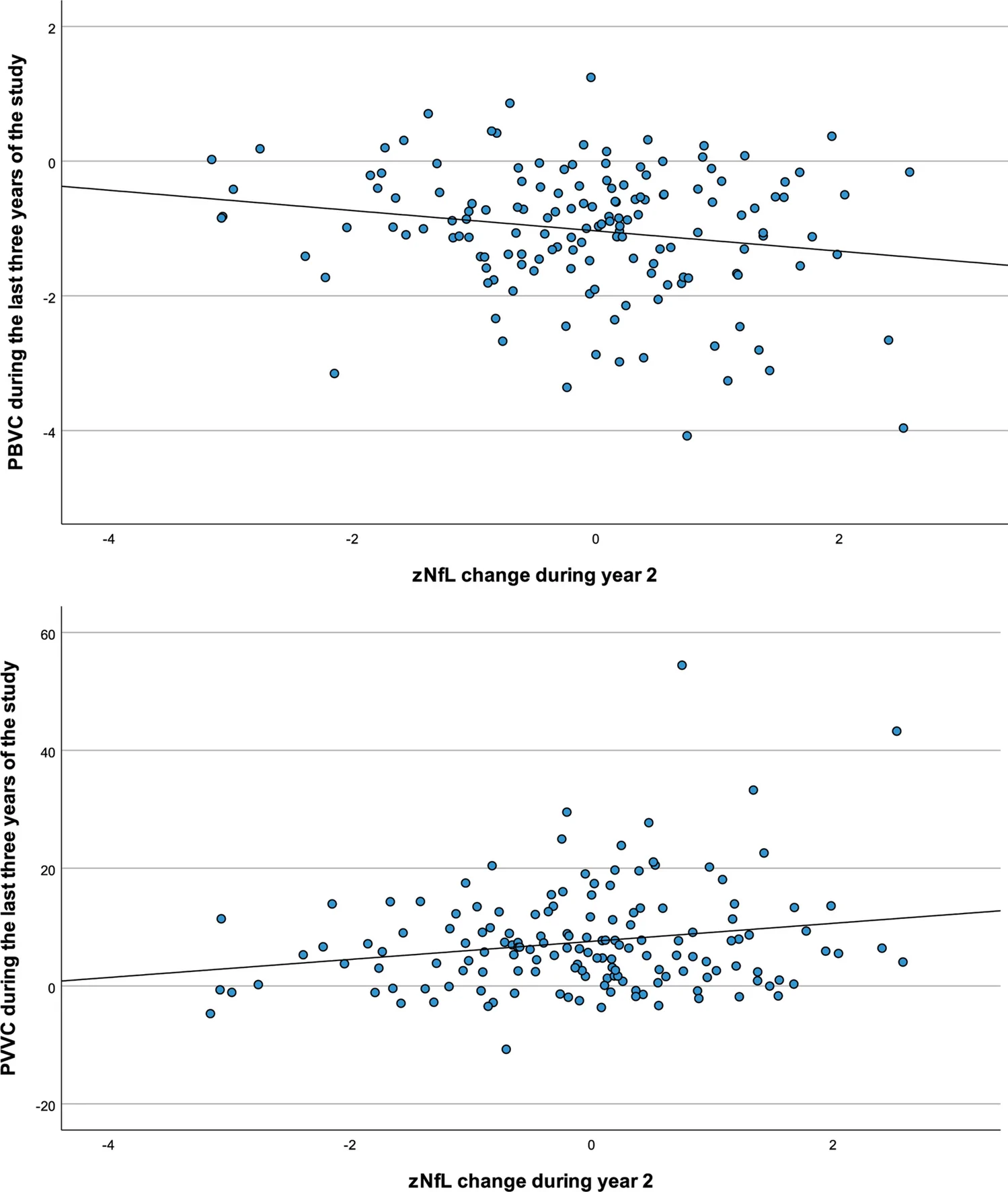
Relationship between changes in Z-scored serum neurofilament light chain levels during year 2 and atrophy during the last three years of the study. Scatterplots showing the relationship between z-scored serum neurofilament light chain (*zNfL*) change during year 2 and percentage brain volume change (*PBVC*; global atrophy) and percentage ventricular volume change (*PVVC*; central atrophy) during the last three years of the study. This shows that an increase in zNfL during year 2 was related to faster atrophy (indicated by a negative PBVC and positive PVVC value) during the last three years of the study

zNfL change during year 1 was not significantly related to atrophy during the last three years of the study. As shown in Fig. 2, increasing zNfL during year 2 was significantly related to faster atrophy later in the study: PBVC_m24-m60_ (*r* = − 0.18, *p* = 0.025), PVVC_m24-m60_ (*r* = 0.20, *p* = 0.015). This was also the case when correcting for baseline MRI and demographic confounders and baseline zNfL.

## Discussion

In this study, we investigated the evolution of age- and BMI-adjusted z-scores of serum concentrations of NfL (zNfL) in the first years of interferon treatment in early MS. Additionally, we investigated how zNfL related to MRI markers of damage and clinical and radiological disease progression. We found that zNfL levels were higher in patients with greater inflammatory activity, but dropped rapidly during the first year of treatment. Cross-sectional baseline zNfL levels reflected lesion burden and were predictive of disease progression but only if MRI measures were not taken into account. We also observed that initial zNfL decreases over time were associated with the resolution of inflammation, while later zNfL increases were associated with a more neurodegenerative profile.

### Baseline zNfL is related to more lesion activity

Higher baseline zNfL levels were related to greater inflammatory activity, in the form of higher lesion loads and the presence of contrast enhancement, as previously also observed [2, 3]. However, in those without contrast enhancement, zNfL levels were also strongly related to T2 lesion volumes, indicating continuing neurodegeneration of axons within pre-existing lesions. This is supported by previous work showing a delay of the peak of sNfL elevation of around 8 weeks after the development of a gadolinium-enhancing lesion [27] and the predictive value of sNfL elevation for progression, suggestive of ongoing neurodegeneration [28]. In line with this, another study has found that chronic white matter inflammation was associated with increased sNfL in patients without gadolinium-enhancing lesions or relapses [29], observed in our study by the correlation of higher baseline zNfL with enlarging lesions during year 1 and year 2.

### Baseline zNfL is related to the resolution of edema

We also found that higher baseline zNfL was related to the amount of lesion shrinkage. We hypothesize that this relationship occurs because more severe neuro(axo)nal damage, as reflected by higher zNfL at baseline, is related to having a more active disease which is accompanied by inflammatory edema which resolves during year 1 after the initiation of treatment. The role of resolving edema is also corroborated by the observation of a positive correlation between baseline zNfL and the volume of resolving edema, quantified in this study as the evolution of baseline gadolinium-enhancing lesions into shrinking lesions during year 1. Earlier work has also shown that lesion shrinkage is related to resolution of inflammation [30]. Interestingly, subjects with pseudoatrophy, classified in our study as showing a substantial amount of atrophy in combination with shrinking lesion activity in the brain during year 1, on average had a higher baseline zNfL compared to those who did not show (substantial) pseudoatrophy. This has not been investigated in other studies before. As such, this could mean that the inflammatory/swollen state of the brain in active disease (given that included subjects recently experienced their first attack) is also related to higher zNfL levels. However, sNfL has been suggested to be related to both inflammation and neurodegeneration [31, 32], denoting the difficulty of pinpointing the exact nature of NfL as a purely inflammatory or neurodegenerative marker.

### Baseline zNfL is related to neurodegeneration

Higher baseline zNfL was related to faster global and central atrophy during year 1 and year 2 and faster later central atrophy, forming an indicator of not only a more neuroinflammatory but also neurodegenerative profile. Although traditionally seen as a marker of acute inflammation, previous studies have shown an association between higher baseline sNfL levels and faster atrophy over 2 to 10 years [3], and especially during periods of inflammatory activity [33], to which this study now adds similar data in very early MS treated with subcutaneous interferon beta-1a. Interestingly, baseline zNfL levels were not related to cross-sectional baseline NBV in our study. This could possibly reflect a delay between lesion activity, sNfL changes, and neurodegeneration, which would explain why there is a relation with later longitudinal change in brain volume. However, our finding is contrary to other research, where cross-sectional higher sNfL levels were associated with a smaller NBV [34]. This discrepancy is possibly related to the phase of the disease, as our study only included subjects with very early MS.

### Baseline zNfL is only predictive if MRI measures are not taken into account

Higher baseline zNfL levels were related to faster time to CDMS, indicating that baseline zNfL is related to faster progression. Additionally, baseline zNfL levels were predictive of both clinical and radiological disease progression. However, once baseline MRI measures and demographics were taken into account, baseline zNfL levels lost their predictive value. This is contrary to another study where sNfL levels were independently associated with conversion to CDMS after adjusting for T2 lesion load and the presence of gadolinium-enhancing lesions (and the presence of oligoclonal bands in CSF) [35]. This discrepancy with previous literature possibly occurs because we corrected for more potential confounders, such as age, previously shown to be related to sNfL levels [36]. We did not find any predictive value of baseline zNfL for EDSS progression, possibly due to the low disability levels and low sample size of people with confirmed progression in this early cohort, as this relation was found in earlier work, although inconsistently [2, 3, 37]. However, we did find predictive value for CDMS, which is consistent with other studies [38]. Future work is still required to investigate the clinical predictive value of sNfL for other measures like cognition and fatigue.

### zNfL levels over time

We found that zNfL levels were highest at baseline, as expected due to the inclusion criterion of recent demyelinating activity, especially in those with more active disease. The levels of zNfL dropped during the first year of the study and stabilized to healthy control levels during the second year of the study, especially for patients with less active disease at baseline, comparable to other observations [39]. These findings suggest that normalization of zNfL may be indicative of the resolution of inflammatory disease activity. This observed decrease was most likely accelerated by the initiation of disease-modifying therapy [40], particularly as treatment was started in patients with recent disease activity. Suppression of inflammatory disease activity following treatment initiation may reduce ongoing axonal injury, resulting in zNfL levels gradually approaching those observed in healthy controls, particularly early in the disease course. Interestingly, in another study zNfL levels remained elevated over time for platform compounds such as interferon [21], possibly due to different treatment strategies. Future studies should now compare these dynamics to more recent and higher efficacy therapies, where faster decreases have been observed [21].

### Associations with longitudinal zNfL changes

In contrast to baseline zNfL results, longitudinal zNfL increases during year 2, i.e., the stable treatment period, were related to later neurodegeneration even after correction for potential confounders. This was not the case for clinical disease progression, possibly due to the early disease stage of this group. Earlier work has been inconsistent, where two studies did not find longitudinal relations for brain atrophy [7, 13] while another study did find that increased sNfL over time was related to worse atrophy [14]. In addition, after the initiation of treatment during year 1, we found that a decrease in zNfL levels during year 1 was related to more lesion shrinkage. In contrast, after correcting for baseline zNfL, an increase in zNfL was related to ongoing disease activity, reflected by new and enlarging lesions (i.e., breakthrough disease activity). The latter effects were very similar to those seen between zNfL increases and new/enlarging lesions during year 2. However, this was not the case in two other studies that showed no relationship between concurrent changes in sNfL and T2LV but a positive correlation with enhancing lesions [7, 14], indicating that acute but not ongoing disease activity was related to changes in sNfL levels over time. Other studies only showed a weak association between percentage sNfL change and change in T2LV [41] and number of T2 lesions [13]. However, our method to quantify lesion volume changes [23] takes longitudinal changes into account and we used z-scores adjusted for age and BMI, which is perhaps needed to increase sensitivity and detect a relationship between changes in sNfL and in T2 lesion load, instead of absolute sNfL concentrations.

### Limitations

This study has several limitations. Measuring/detecting resolving edema and pseudoatrophy by respectively quantifying enhancing sections of shrinking lesions and using a literature-based cutoff in combination with median change in shrinking lesion volume is explorative in nature and assumes that enhancing lesions indicate edema. Although pseudoatrophy is more pronounced in patients with active inflammation [42], advanced imaging methods like myelin water fraction imaging and high-resolution FLAIR are needed for better definition. Future studies, especially in patients on high-efficacy treatments, are needed since pseudoatrophy effects may be more pronounced [43]. Such studies should also include information on relapses and spinal cord lesions, which was unavailable for the current study. Because early initiation of disease-modifying therapies are now the standard of care, we did not include the delayed treatment group. Additionally, the specific design of the delayed treatment arm would complicate the interpretation of the data and result in a large increase in the number of tests. Our results apply at the group level and cannot be generalized to individuals and other treatment strategies as well as progressive disease. For instance, a recent study identified specific phenotypes describing the relationship between sNfL and MRI in a larger more heterogeneous population, including progressive MS [44]. To establish generalizability, large-scale, longitudinal studies are required to validate sNfL as a routine clinical biomarker. Using BMI- and age-adjusted z-scores instead of absolute sNfL is more accurate [21], but MS-specific reference databases could further improve accuracy. Since only sNfL was measured, our findings should be replicated for CSF NfL, which generally correlates strongly with serum but is higher in concentration [1], and additional biomarkers beyond NfL should be investigated.

## Conclusion

In subjects with early MS starting interferon treatment, zNfL levels at baseline were predictive of disease progression. However, this predictive value was lost when taking MRI measures into account, likely due to the strong relationship between zNfL and lesion activity. Therefore, baseline zNfL reflects inflammatory disease activity and seems especially useful in the absence of MRI. An increase in zNfL over time was linked to both less lesion shrinkage (possibly reflecting less resolution of edema) as well as more active disease and subsequent faster atrophy. Both MRI and NfL have a unique role in the prediction and monitoring of progression in MS.

## Supplementary Information

Below is the link to the electronic supplementary material.

Supplementary file1 (PDF 127 kb)
