# Supplementary material for: The predictive value of serum neurofilament light chain levels beyond MRI measures for clinical and radiological disease progression in the earliest stages of multiple sclerosis
Source: J Neurol. 2026 Sep 9;273(10):584. doi: 10.1007/s00415-026-14100-9 (PMC13558367; doi:10.1007/s00415-026-14100-9)
Supplement: Supplementary file 1 [file 415_2026_14100_MOESM1_ESM.pdf]

Supplementary Material

Participating Centers

Argentina
Mendoza, Argentina

Australia
Sydney, Australia

Austria
Graz, Austria
Innsbruck, Austria

Belgium
B-Leuven, Belgium
Bruges, Belgium

Bulgaria
Pleven, Bulgaria
Rousse, Bulgaria
Shumen, Bulgaria
Sofia, Bulgaria
Varna, Bulgaria

Canada
Montreal, Quebec, Canada
Ontario, Canada
Victoria British Columbia, Canada

Croatia
Karlovac, Croatia
Osijek, Croatia
Rijeka, Croatia
Split, Croatia
Zagreb, Croatia

Czechia
Hradec Králové, Czechia
Olomouc, Czechia
Prague, Czechia

Estonia
Tallinn, Estonia
Tartu, Estonia

Finland
OYS, Finland
Vantaa, Finland

France
Paris, France
Poissy, France

Germany
Hanover, Germany
Henningsdorf, Germany
Munich, Germany

Greece
Athens, Greece

Israel
Ness Ziona, Israel
Safed, Israel
Tel Litwinsky, Israel

Italy
Catania, Italy
Milan, Italy
Padova, Italy
Roma, Italy

Latvia
Riga, Latvia

Lebanon
Beirut, Lebanon

Morocco
Rabat, Morocco

Poland
Bialystok, Poland
Lodz, Poland
Warsaw, Poland
Wroclaw, Poland

Portugal
Lisbon, Portugal

Romania
Bucharest, Romania
Iași, Romania
Târgu Mureş, Romania
Timișoara, Romania

Russia
Moscow, Russia
Nizhny Novgorod, Russia
Novosibirsk, Russia
Saint Petersburg, Russia
Samara, Russia
Saratov, Russia
Yekaterinburg, Russia

Saudi Arabia
Riyadh, Saudi Arabia

Serbia
Belgrade, Serbia
Niš, Serbia

Slovakia
Prešov, Slovakia

Spain
Barcelona, Spain
Bilbao, Spain
Madrid, Spain
Seville, Spain

Turkey (Türkiye)
Istanbul, Turkey (Türkiye)
